# The Combustion Behaviors and Flame-Retardant Mechanisms of Cu Coating as Protection for Titanium Alloys

**DOI:** 10.3390/ma19050944

**Published:** 2026-02-28

**Authors:** Jianjun Li, Shujing Wang, Pengfei Jin, Cheng Zhang, Congzheng Wang

**Affiliations:** 1 National Key Laboratory for Advanced Metals and Materials, University of Science and Technology Beijing, Beijing 100083, China; lijjustb@163.com (J.L.); wsjtyut@126.com (S.W.); mynameisjinpengfei@outlook.com (P.J.); 2 Civil Aviation Safety Engineering Institute, Civil Aviation Flight University of China, Guanghan 618307, China

**Keywords:** Cu coatings, flame-retardant coatings, metal combustion, titanium fire

## Abstract

**Highlights:**

**What are the main findings?**

- Proposes using a highly conductive copper coating to enhance titanium alloy flame retardancy
- Establishes a link between coating factor and critical ignition conditions for titanium alloys
- Identifies a (Ti_0.5_Al_0.5_)Cu intermetallic compound with an “anchoring” effect on flame retardancy

**What are the implications of the main findings?**

- Provide a practical potential surface-engineering method for safer titanium alloy use
- Deliver key data and insight for flammable metallic materials research
- Lay the groundwork for studying interfacial reaction-controlled combustion suppression

**Abstract:**

This study investigates the influence of highly thermally conductive coatings on the combustion thresholds of a TC4 titanium alloy, aiming to address the flame-retardant protection requirements for titanium alloys. The findings reveal that, in terms of combustion thermodynamics, as the thickness of the copper coating increases from 100 μm to 300 μm, the critical ignition power rises by 125–170 W compared to the substrate (235 W). Additionally, the critical oxygen pressure increases by 0.21–0.51 MPa relative to the substrate (0.03 MPa), and the ignition temperature is elevated by 119–184 K above that of the substrate (848.80 K). This phenomenon is primarily due to the high thermal diffusivity of copper. Increased coating thickness further enhances heat dissipation, significantly suppressing the local heat accumulation rate and thereby improving the coating’s combustion resistance. In terms of combustion kinetics, under fixed experimental conditions, the copper coating extends the ignition delay time by 0.670 s and reduces the combustion propagation rate by approximately 21% compared to the substrate (26.772 mm/s). The post-combustion microstructural analysis indicates that during the reaction process, the copper coating forms a TiCu_2_Al-type intermetallic compound (Ti_0.5_Al_0.5_)Cu. This structure exerts an “anchoring” effect on the substrate material, decreases the Ti/O reaction efficiency, and consequently achieves effective flame retardancy. These findings inform the subsequent design and optimization of copper-based abradable coatings with enhanced combustion resistance.

## 1. Introduction

In the field of aero-engines, to achieve higher thrust-to-weight ratios and operational efficiency, high-strength, lightweight titanium alloys are widely used in core components such as high-pressure compressors [1,2,3,4]. However, titanium alloys are prone to “titanium fire” in high-temperature and oxygen-enriched environments. Their intense exothermic combustion characteristics can rapidly ablate components, posing a serious threat to engine safety [5,6,7], which has become a key bottleneck restricting the application of titanium alloys under extreme operating conditions.

Engineers commonly employ abradable sealing coatings to enhance the durability and environmental service performance of compressor components [8,9,10]. Such coatings actively control the clearance between rotating and stationary parts through an abradable design, reducing gas leakage under high-temperature and high-speed conditions, thereby significantly improving mechanical efficiency and operational performance [8,11,12,13,14]. It is noteworthy that copper materials already have a practical application foundation in the field of titanium alloy protection, primarily in the form of Cu-based abradable coatings for scenarios such as engine-seal clearance control [13,15,16]. However, existing research has predominantly focused on optimizing their abradability to suit friction and wear conditions, while systematic studies on the fire-resistant performance of such Cu-based coatings are extremely scarce, hindering their further application in “titanium fire” protection.

Given Cu’s high melting point (~1083 °C) and superior thermal conductivity (401 W·m^−1^·K^−1^ at room temperature) [17,18,19], combined with the formation of dense CuO/Cu_2_O oxide films at elevated temperatures [20,21,22] and a notable interfacial compatibility with titanium alloys, these properties render it a highly promising matrix material for titanium alloy flame-retardant coatings. Although literature specifically on Cu coating’s flame retardancy is limited, insights can be drawn from studies on copper-modified titanium. For example, research has shown that adding copper to pure titanium promotes Ti_2_Cu formation, which improves flame retardancy [23]. The mechanism involves two key processes during combustion: the segregation of liquid Ti_2_Cu at the solid–liquid interface hinders oxygen diffusion, and the presence of Ti_2_Cu at grain boundaries absorbs heat, both of which help suppress combustion propagation. Compared with developing new alloys, fabricating coating structures tailored to service environments on existing components is more economical. The high ductility of a Cu coating can enhance the interfacial adhesion with titanium alloy substrates [24,25,26], and its excellent thermal conductivity can mitigate thermal expansion mismatch issues, offering potential solutions to the common problem of high-temperature cracking and spalling in existing protective coatings [27,28,29,30], thereby providing a new technical pathway for titanium alloy “titanium fire” prevention. Currently, to advance the application of Cu-based coatings in titanium alloy flame-retardant protection, two core issues must be addressed urgently: first, systematically investigating the influence of copper coatings on the combustion thresholds (e.g., ignition temperature and critical oxygen pressure) of titanium alloys, as these thresholds are key indicators for evaluating whether coatings can delay or suppress ignition; and second, deeply elucidating the Ti/O combustion reaction mechanisms and microstructural evolution under the protection of copper coatings to clarify their anti-burning mechanisms, which is of great significance for guiding the design and development of next-generation copper-based abradable coatings.

To address these issues, this study utilized a self-developed laser ignition test system to systematically investigate the influence of copper coatings on the combustion threshold values of a TC4 titanium alloy. Furthermore, characterization techniques such as SEM, EDS, and XRD were employed to analyze the compositional and microstructural evolution of specimens during combustion, elucidating the regulatory mechanisms of copper coatings on the Ti/O combustion reaction process. The aim was to provide reliable data support and a theoretical basis for the optimized design of copper-based anti-burning coatings and their application in preventing titanium alloy ignition.

## 2. Materials and Methods

The substrate material employed was a TC4 alloy (Ti-6Al-4V), supplied by BAOTI Group Co., Ltd., Shaanxi, China. The alloy was cut into 4 × 4 × 35 mm prismatic specimens, followed by mechanical polishing with 1000-grit sandpaper prior to coating deposition. Commercially available Cu powder (Beijing Yanbang New Material Technology Co., Ltd., Beijing, China) was selected as the feedstock material. The Cu coating was deposited using a high-velocity oxygen-fuel (HVOF) spraying system (DJ-2700, Oerlikon Metco, Wohlen, Switzerland), with the specific process parameters listed in Table 1. The Cu coating was deposited only on one of the 4 mm × 35 mm faces of the TC4 substrate, and this entire face was fully covered by the coating. The resulting coating thickness was controlled within the range of 100–300 μm.

Ignition testing was performed under a set of laser ignition test systems following the methodology described in [31], with critical ignition conditions defined according to established criteria [32]. Prior to testing, the combustor was evacuated to a vacuum level of 10–2 Pa. A MCS640 infrared thermometer (MCS640, LUMASENSE TECHNOLOGIES, Milpitas, CA, USA, with an accuracy of ±5 K) was utilized to monitor the sample temperature throughout the combustion process, and the ignition temperature was identified as the abrupt inflection point on the resulting temperature–time curve. The emissivity values of the Cu coating, determined via concurrent calibration with a two-color pyrometer (M322, Bavaria, Germany), were fixed at 0.80 for all infrared temperature measurements. Simultaneously, a high-speed camera (Pco.dimax S4, Kelheim, Germany) was used to record the ignition and combustion events for visual characterization of the combustion phenomena. The infrared thermometer and high-speed camera were synchronized to ensure precise temporal correlation between the temperature data and visual observations.

To facilitate a qualitative comparison of the microstructural evolution among the different coatings after combustion, the post-combustion samples were preserved through argon-quenching. These specimens were sectioned longitudinally and then ground, polished, and etched in a 1:3:6 HF:HNO_3_:H_2_O solution for subsequent microstructural characterization. A microstructural analysis was performed using a scanning electron microscope (SEM, JEOL JSM 7200F, Tokyo, Japan) equipped with an energy-dispersive spectrometer (EDS). Phase composition was identified by X-ray diffraction (XRD, Bruker D8 Focus, Billerica, MA, USA) with Cu Kα radiation at a scanning rate of 2°/min. Coating porosity was evaluated from backscattered electron images using ImageJ 1.8.0 software. The porosity was calculated using a thresholding and binary segmentation approach, where the porous areas were identified by adjusting the grayscale threshold and then measured as a percentage of the total coating area in each image. A total of five BSE images were analyzed, taken from three representative locations across the coated surface (center and both ends) to ensure statistical representativeness.

## 3. Results

### 3.1. The Microstructures and Compositions of the Coatings

**Figure 1:**
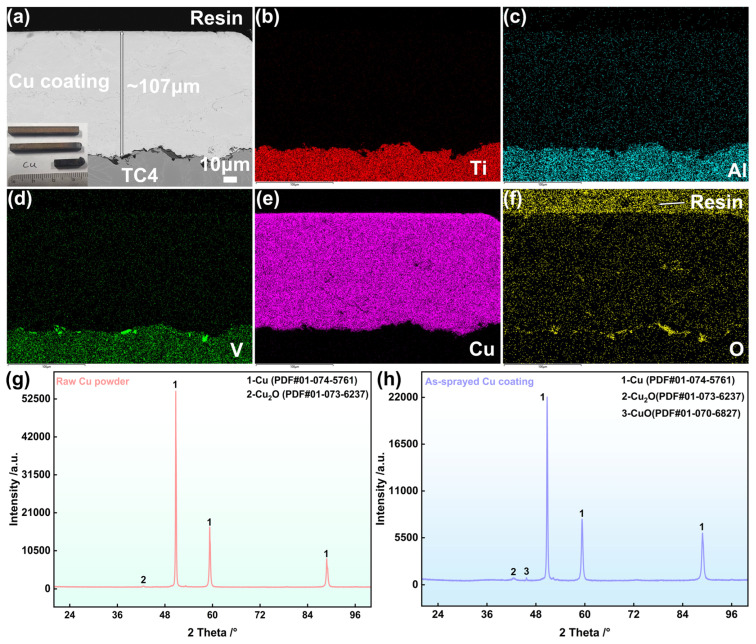
Microstructure of the as-sprayed copper coating sample (**a**), compositions distributions (**b**–**f**), XRD result of Cu feedstock (**g**), and XRD results of the as-sprayed coating (**h**).

Figure 1a illustrates the microstructural characteristics of the as-sprayed Cu coating, possessing a dense structure. The EDS mapping results of the coating cross-section (Figure 1b–e) indicated no significant elemental interdiffusion at the coating–substrate interface. Furthermore, the oxygen distribution shown in Figure 1f reveals no apparent oxygen ingress, suggesting an extremely low level of oxidative contamination during the spraying process. The XRD pattern of the Cu feedstock was primarily the pure Cu phase (PDF#01-074-5761), with only minimal traces of Cu_2_O (Figure 1g). After spraying, the coating remained predominantly composed of the pure Cu phase, and minor amounts of Cu_2_O (PDF#01-073-6237) and CuO (PDF#01-070-6827) were detected due to exposure to high temperatures and oxygen in the atmosphere (Figure 1h). However, the diffraction peaks’ low intensity suggests that the presence of these oxide phases was insignificant and had no substantial impact on the overall composition of the coating.

### 3.2. Combustion Behaviors of the Coatings

**Figure 2:**
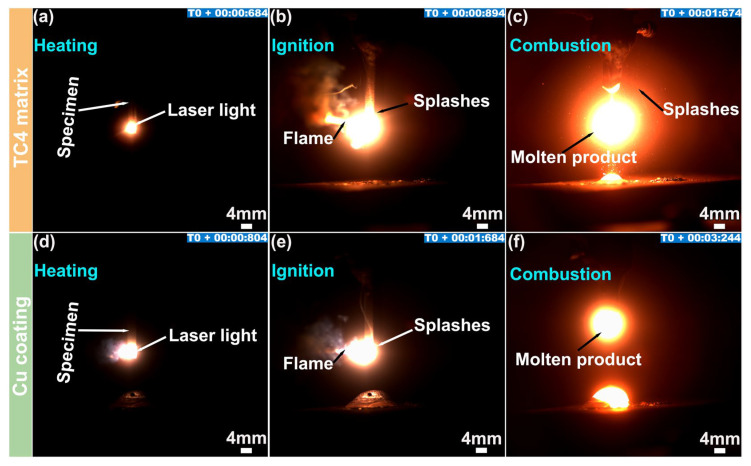
Combustion behaviors of the TC4 titanium alloy sample (**a**–**c**) and the Cu coating specimen (**d**–**f**) under the same conditions.

Figure 2 displays the combustion behaviors of the Cu coating samples and TC4 titanium substrate samples under the conditions of an oxygen pressure of 0.30 MPa, a laser power of 480 W, and room temperature. Figure 2a–c depicts the combustion process of the bare TC4 substrate, which could be divided into three stages: laser heating (Figure 2a), ignition (Figure 2b), and stable combustion (Figure 2c). During the heating stage, a bright, nearly circular spot appeared in the laser-irradiated zone. At the ignition moment, the brightness increased sharply, accompanied by flames, a splattering of the material, and the emission of smoke. Upon stable combustion, the products periodically dripped in a near-ellipsoidal form under gravity, along with liquid splashes. By comparing the timestamp in Figure 2a,b, it was observed that the TC4 substrate was ignited after a short laser exposure, with a delay time (Δt) of only 0.210 s. It then transitioned into a stable combustion stage and was nearly fully consumed approximately Δt = 1.307 s after ignition (Figure 2c). These results indicated that the TC4 titanium alloy ignited rapidly under laser irradiation and sustained an exceptionally high combustion rate of approximately 26.772 mm/s, confirming its inherent combustion sensitivity and rapid-burning nature.

The combustion process of the Cu-coated sample, as shown in Figure 2d–f, is similar to that of the bare TC4 substrate. However, a notably delayed ignition response was observed. Instead of igniting immediately upon laser exposure, ignition commenced after a delay of Δt = 0.880 s (Figure 2e), which was 0.670 s longer than that of the bare substrate. After ignition, the Cu coating sample was completely consumed within approximately Δt = 1.660 s (Figure 2f), corresponding to a combustion propagation rate of approximately 21.084 mm/s. These results demonstrated that the Cu coating effectively reduced the ignition sensitivity, prolonged the ignition delay, and slowed the combustion propagation of the TC4, confirming its distinct flame-retardant function.

**Figure 3:**
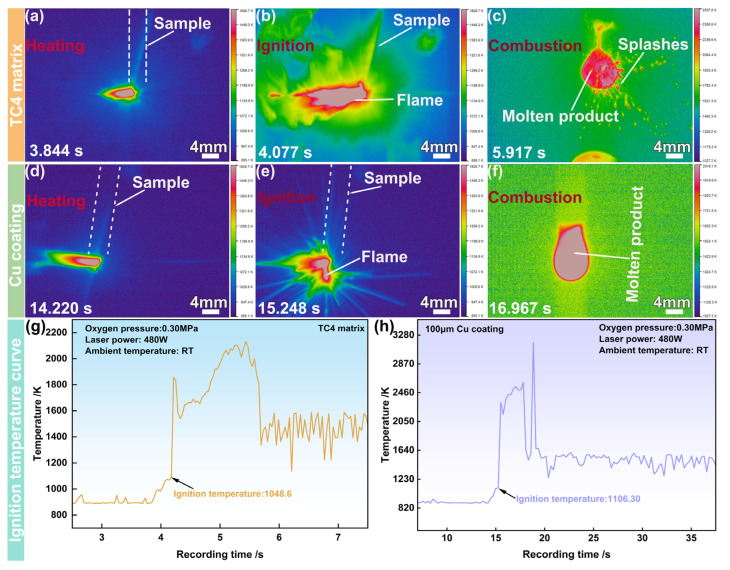
Temperature distribution during the combustion process of the TC4 titanium alloy sample (**a**–**c**), and the Cu coating sample (**d**–**f**), temperature curve of the TC4 alloy sample (**g**), and temperature curve of the Cu coating sample (**h**).

Figure 3 illustrates the temperature-field evolution of the synchronous test in Figure 2. For the TC4 substrate (Figure 3a–c), at the ignition moment, the temperature-field distribution expanded instantaneously (Figure 3a,b). Then, it entered the stable combustion stage, and the temperature of the molten product approached 2537.60 K (Figure 3c). For the Cu coating sample (Figure 3d–f), similar phenomena could be observed over the heating stage to the ignition moment compared to the TC4 substrate (Figure 3d,e). However, during the stable combustion stage, the melt temperature remained at approximately 2019.10 K (Figure 3f), which was substantially lower than that of the bare TC4 substrate, demonstrating the effective suppression of combustion intensity. Further, the ignition temperature values for the bare TC4 and Cu coating samples could be determined to be 1048.6 K and 1106.30 K, respectively, indicating that the Cu coating elevated the ignition temperature by 57.70 K (Figure 3g,h).

### 3.3. The Critical Ignition Conditions of the Coatings

**Figure 4:**
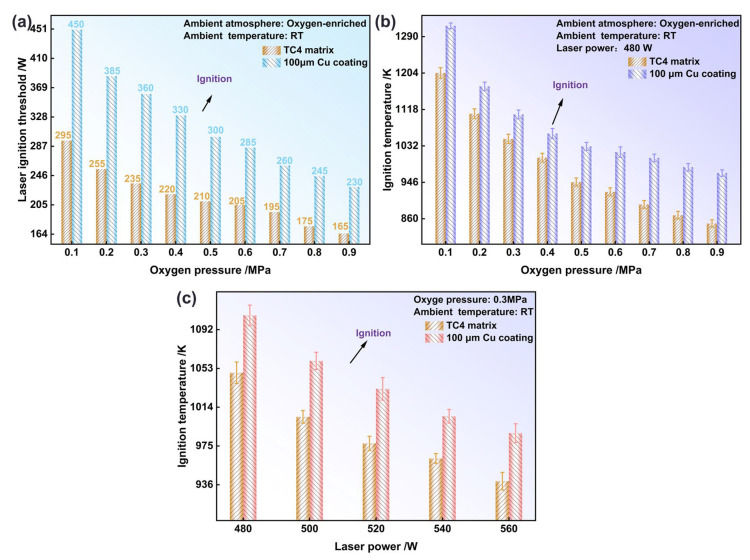
The critical conditions for ignition of the Cu coating samples against the TC4 substrate samples: the critical ignition power as a function of oxygen pressure (**a**), the variations in ignition temperature with oxygen pressure (**b**), and the relationship between ignition temperature and laser power (**c**).

Figure 4a compares the laser ignition threshold as a function of oxygen pressure for the Cu-coated sample and the bare TC4 substrate. The critical power was observed to decrease gradually for both materials as oxygen pressure was increased from 0.10 to 0.90 MPa. However, a consistently higher power requirement was maintained by the coated sample across the entire pressure range. For instance, at 0.10 MPa, 450 W was required for the coated sample, which was 165 W above the 295 W needed for the bare substrate. Even at 0.90 MPa, the critical power of the coated sample (230 W) remained 75 W higher than that of the substrate (165 W). These results confirmed that the energy-input threshold for ignition was significantly raised by the Cu coating. In Figure 4b, the ignition temperature is presented as a function of oxygen pressure under a fixed laser power of 480 W. The ignition temperatures of both materials were reduced as oxygen pressure was increased from 0.10 to 0.90 MPa. Over this pressure range, an ignition-temperature advantage of 57.70–119.10 K was maintained by the coated sample relative to the bare substrate.

Figure 4c shows the relationship between the ignition temperature and laser power at a fixed oxygen pressure of 0.30 MPa. The ignition temperature was lowered for both materials when the laser power was increased from 480 to 560 W, indicating a reduced energy threshold for ignition. At 480 W, ignition occurred at 1106.3 K for the coated sample, which was 85.70 K higher than the 1048.60 K recorded for the substrate. At 560 W, the temperatures were reduced to 987.90 K and 939.60 K, respectively, narrowing the difference to 48.30 K. This demonstrated that while the ignition temperature was consistently elevated by the coating, its protective margin was diminished at higher laser powers. According to previous work [31], this behavior can be attributed to an enhanced laser–material interaction at higher powers, which increases the surface energy-deposition rate and accelerates thermal accumulation. Once a critical level of energy per unit time is exceeded, ignition is enabled at a lower bulk temperature; that is, the ignition temperature decreases as the energy-input rate is increased.

**Figure 5:**
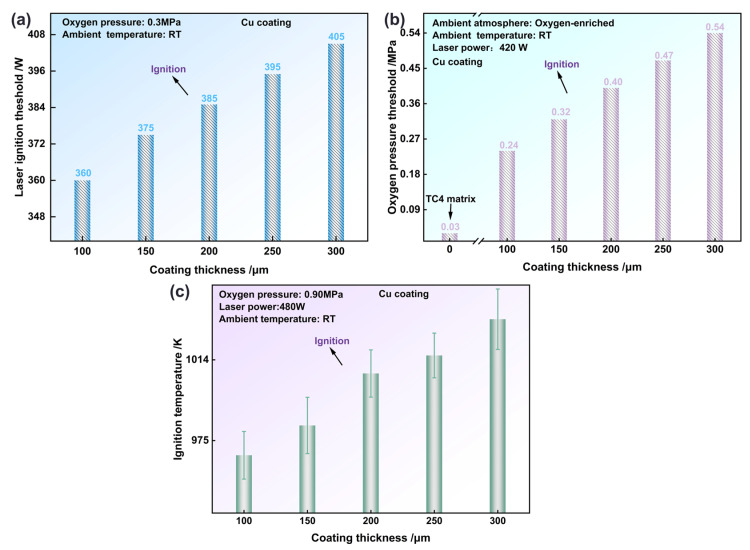
The effects of coating thickness on critical ignition conditions: laser ignition threshold (**a**), oxygen pressure threshold (**b**), and ignition temperature (**c**).

Figure 5a illustrates the correlation between the Cu coating thickness and laser ignition threshold at an oxygen pressure of 0.30 MPa. As the coating thickness was increased from 100 μm to 300 μm, the laser ignition threshold was observed to rise steadily from 360 W to 405 W. Compared to the TC4 substrate (235 W), the Cu coating raised the critical power by 125–170 W, indicating a clear thickness-dependent enhancement of the ignition threshold. Figure 5b shows the influence of coating thickness on the critical oxygen pressure. The critical oxygen pressure followed an approximately parabolic growth with increasing thickness, rising from 0.24 MPa at 100 μm to 0.54 MPa at 300 μm. Relative to the TC4 substrate (0.03 MPa), the coating elevated this threshold by 0.21–0.51 MPa, effectively delaying oxygen diffusion to the substrate interface and, thus, improving ignition resistance under oxygen-enriched conditions. Under a fixed laser power (480 W) and oxygen pressure (0.90 MPa), the effect of coating thickness on ignition temperature is presented in Figure 5c. As thickness increased from 100 μm to 300 μm, the ignition temperature rose in a near-exponential manner, from 967.90 K to 1033.60 K. Compared to bare TC4 (848.80 K), the coated samples exhibited an increase of 119–184 K in ignition temperature, confirming that the thicker coatings provided stronger protection in high-temperature, high-pressure oxygen environments.

### 3.4. Microstructure Analysis of the Post-Ignited Samples

**Figure 6:**
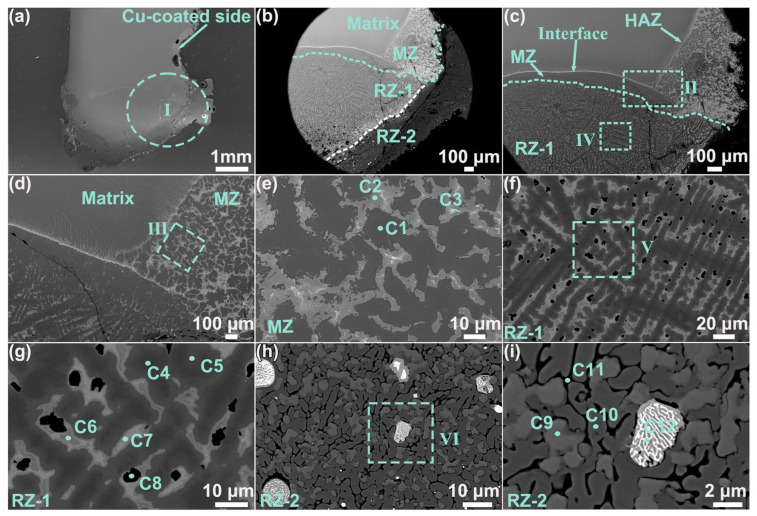
Microstructure of the post-combustion Cu coating sample: low-magnification images (**a**–**c**), high-magnification images of the MZ (**d**,**e**), high-magnification images of RZ-1 (**f**,**g**), and high-magnification images of RZ-2 (**h**,**i**).

Figure 6 presents the post-combustion microstructure of the copper-coated sample. A low-magnification SEM image showing the overall morphology is provided in Figure 6a. A selected characteristic region (Region I in Figure 6a) is further displayed in Figure 6b,c, where the microstructure is divided into several zones: the matrix, heat-affected zone (HAZ), melting zone (MZ), and reaction zones (RZ-1 and RZ-2). A higher magnification view shown in Figure 6c delineates these zones. The high-magnification image of the MZ (Region II in Figure 6c) is detailed in Figure 6d,e. Figure 6d shows that the MZ consisted of two distinct parts: one comprising the largely molten TC4 substrate and the other a mixed melting region of the coating and substrate materials. A higher magnification image of the mixed region (Region III in Figure 6d, as shown in Figure 6e) revealed that there were three distinct phases, which are labeled C1, C2, and C3 in the MZ.

Regarding RZ-1, a local detail image (Region IV in Figure 6c), as illustrated in Figure 6f, showed that the microstructure consisted of a black dendritic structure, a mixed lamellar structure of black and light-gray phases, and a deep black granular phase. According to Figure 6g (Region V in Figure 6f), the black dendrites mainly consisted of phases C4 and C5, the light-gray structure was primarily composed of phases C6 and C7, and the granular phase C8 was predominantly attached to the periphery of the mixed phases C6 and C7. The local microstructure of RZ-2 is shown in Figure 6h. There are four distinct phases according to Figure 6i (Region VI in Figure 6h), and they are labeled as C9~C12.

**Figure 7:**
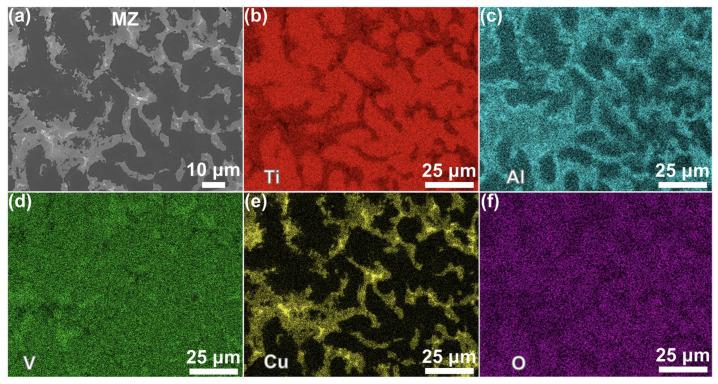
EDS element distribution of the MZ: (**a**) the image of Figure 6e, (**b**) Ti, (**c**) Al, (**d**) V, (**e**) Cu, and (**f**) O.

Figure 7 presents the EDS elemental mapping results for the MZ (Figure 6e). Phase C1 was shown to be primarily enriched in Ti and O (Figure 7b,f), with contents of 73.84 at% and 24.60 at%, respectively. With reference to the Ti-O binary phase diagram [27], Phase C1 was identified as molten α-Ti derived from the TC4 substrate. Phases C2 and C3 were mainly enriched in Cu and Al, with minor V (Figure 7c,e). According to Point C2 in Table 2, Phase C2 consisted of 30.04 at% Ti, 23.11 at% Al, and 33.90 at% Cu, indicating a mixture of Ti, Al, and Cu. Phase C2 could be interpreted as a mixture of copper and β-Ti. In contrast, Phase C3 contained 80.12 at% Cu (Point C3 in Table 2), suggesting that it consisted mainly of a residual Cu coating material adhered to Phase C2.

**Figure 8:**
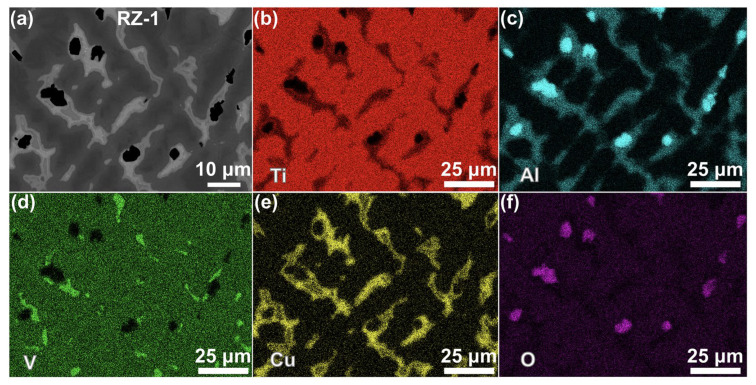
EDS elemental results distribution of RZ-1: (**a**) the image of Figure 6i, (**b**) Ti, (**c**) Al, (**d**) V, (**e**) Cu, and (**f**) O.

Figure 8 presents the EDS mapping results for the reaction zone RZ-1. In this region, Phases C4 and C5 were primarily enriched in Ti and O (Figure 8b,f). According to Point C4 in Table 2, the composition was measured as 64.48 at% Ti and 33.51 at% O, indicating that Phase C4 consisted of α-Ti. Similarly, Phase C5 (Point C5 in Table 2) contained 51.22 at% Ti and 47.52 at% O, corresponding to a Ti:O atomic ratio close to 1:1, which identified it as TiO. Consistent with previous studies on titanium-alloy combustion [4,28], the formation of these phases could be attributed to a peritectic reaction between the liquid phase and the α-Ti during combustion (L + α-Ti → TiO). Phase C6 was found to be enriched mainly in V (67.54 at%), with minor Ti (16.23 at%) and Cu (8.33 at%) (Figure 8d), suggesting it represented segregated metallic V from the TC4 alloy. Phase C7 was characterized by Ti (25.51 at%), Al (22.61 at%), and Cu (50.50 at%) contents (Point C7 in Table 2), with an atomic ratio approximating 1:1:2, corresponding to a (Ti,Al) Cu_2_-type intermetallic phase [33] with a measured thickness of approximately 2–4 μm. Phase C8 was composed predominantly of Al (43.95 at%) and O (54.04 at%), providing an Al:O ratio near 2:3, which identified it as Al_2_O_3_.

**Figure 9:**
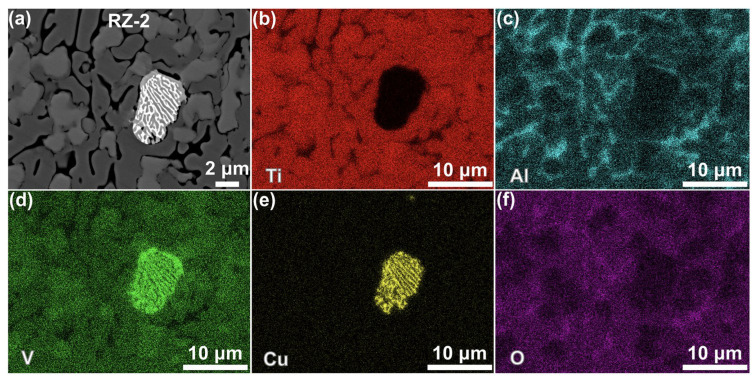
EDS elemental distribution in RZ-2: (**a**) the image of Figure 6i, (**b**) Ti, (**c**) Al, (**d**) V, (**e**) Cu, and (**f**) O.

Figure 9 presents the EDS mapping results for the reaction zone RZ-2. Phases C9 and C10 were mainly enriched in Ti and O. Phase C9 consisted of 51.88 at% Ti and 41.58 at% O (Point C9 in Table 2), corresponding to a Ti:O atomic ratio close to 1:1, thus identifying it as TiO. Similarly, Phase C10 showed contents of 38.57 at% Ti and 55.91 at% O), exhibiting a Ti:O ratio near 2:3, and it could be determined to be Ti_2_O_3_. Phase C11 was enriched in Al and O (Figure 9c,f), and its compositions yielded an Al:O ratio approximating 2:3 (Point C11 in Table 2) and confirmed as Al_2_O_3_. Phase C12 contained 59.43 at% Cu and 27.23 at% V, with negligible oxygen, and it was therefore interpreted as a mixture of the residual coating Cu and segregated V element. It should be noted that, in fact, the RZ-2 area was the combustion reaction product of the pure TC4 matrix. This was attributed to the sample design, in which only one side was copper-coated while the others remained exposed, a configuration that aligned with the unidirectional laser irradiation and simplified specimen preparation.

**Figure 10:**
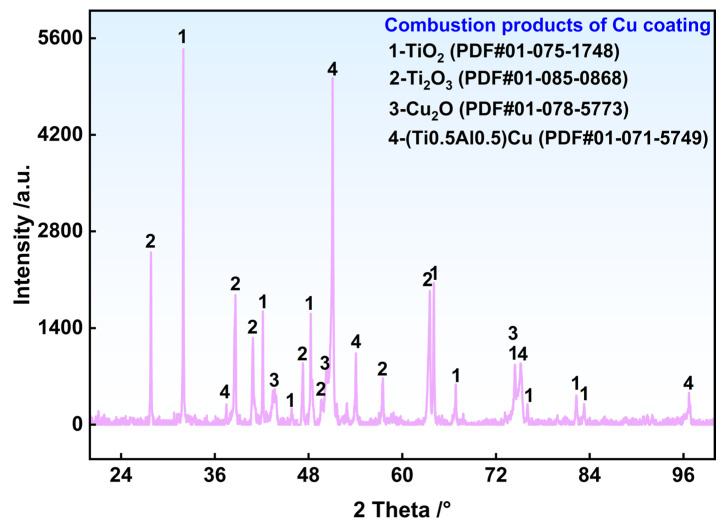
XRD results of the post-combustion slag of the Cu sample.

Under identical conditions of 0.30 MPa oxygen pressure and 480 W laser power, a Cu coating sample was subjected to a test. The fully combusted product was collected and ground into powder for XRD phase identification. As shown in Figure 10, the combustion products consisted mainly of TiO_2_, Ti_2_O_3_, Cu_2_O, and (Ti_0.5_Al_0.5_)Cu, which was consistent with the EDS analysis results. The TiO_2_ and Ti_2_O_3_ were derived primarily from the further oxidation of Phase C5 (TiO) in the reaction zone RZ-1 and Phase C9 (TiO) in RZ-2, while Phase C10 (Ti_2_O_3_) in RZ-2 remained stable under the given temperature and oxygen pressure. The Cu_2_O originated partly from any pre-existing Cu_2_O in the original coating and mainly from the oxidation of Phase C3 in the melting zone (MZ) and Phase C12 in RZ-2. The compound (Ti_0.5_Al_0.5_)Cu matched the Phase C7 in RZ-1. Although distinct diffraction peaks for Al- or V-related oxides were not observed in the XRD pattern, this absence may be attributed to the random sampling of the powder for the XRD analysis, which could cause Al- and V-based oxides to fall below the detection limit of the XRD measurement.

## 4. Discussion

The copper coating enhanced the combustion resistance of the TC4 titanium alloy through dual thermal and kinetic mechanisms. Thermally, the high thermal diffusivity of the copper promoted rapid heat dissipation from the substrate, suppressing local heat accumulation. As the coating thickness increased, this effect became more pronounced, significantly raising the critical ignition power, critical oxygen pressure, and ignition temperature. Kinetically, during combustion, the coating reacted with the substrate to form a TiCu_2_Al-type intermetallic compound (e.g., (Ti_0.5_Al_0.5_)Cu).This continuous TiCu_2_Al-type intermetallic compound layer formed at the Cu–Ti interface, as evidenced by the BSE cross-sectional image (Figure 6g) and the corresponding EDS point analyses (Figure 8e), acting as having a role in “anchoring” the Ti element and effectively impeding direct contact between oxygen and the Ti substrate, thereby limiting Ti diffusion and significantly reducing the efficiency of the Ti–O reaction. While prior work on flame-retardant coatings for titanium alloys has mainly focused on passive thermal barriers or ablation-resistant layers, the present study demonstrated for the first time that a thermally conductive Cu coating could simultaneously act as a heat sink and form a continuous Ti–Cu intermetallic reaction layer that chemically anchored the substrate and kinetically suppressed the Ti–O reaction.

Based on the described dual thermal and kinetic mechanisms by which the copper coating enhanced the combustion resistance of the TC4 titanium alloy, future research directions may focus on the below-discussed aspects to further advance the understanding and application of this flame-retardant approach.

First, regarding coating optimization and design, efforts should be directed toward developing composite and multilayer coating systems. For instance, alloying elements such as nickel or dispersed ceramic phases can be incorporated into a copper matrix to synergistically improve the coating’s thermal barrier effect, high-temperature stability, and oxygen diffusion barrier capability. Systematic research is needed to design compositional gradients, thickness distributions, and interfacial structures of the coatings. This would aim to ensure efficient heat dissipation and reaction inhibition while optimizing the mechanical compatibility between the coating and the substrate, thereby preventing the spallation caused by thermal expansion mismatch or excessive formation of brittle phases.

Second, in terms of preparation processes and engineering implementation, emphasis should be placed on achieving coating uniformity, adhesion strength, and coverage on components with complex geometries. Comparative studies should be conducted on different deposition techniques, such as cold spray, laser cladding, and magnetron sputtering, to evaluate their effects on coating microstructure, defect density, and flame-retardant performance. Quantitative relationships between process parameters, coating microstructure, and protective properties should be established.

Third, concerning validation under realistic environments and long-term reliability evaluation, future research must extend beyond standard laboratory test conditions. Simulations of multi-field coupled environments, such as high-temperature, high-speed gas-flow erosion and thermal cycling, mechanical vibration, and foreign object impact, as encountered in actual service conditions such as aero-engines, should be performed. This will enable a comprehensive evaluation of the degradation mechanisms and service life of coatings under such dynamic loads. Long-term oxidation experiments, thermal fatigue tests, and residual strength assessments after combustion exposure are crucial for determining the safe operational limits of such coatings.

Finally, at the level of system integration and lifecycle performance assessment, future research should address the potential impact of coating on the overall performance of components. This includes evaluating possible effects on the substrate’s mechanical properties, such as fatigue strength and fracture toughness, and exploring compatibility with damage-tolerant design principles. Moreover, from an engineering application perspective, a comprehensive analysis of coating process cost, efficiency, environmental impact, and component reparability should be conducted to provide systematic evidence for technology transfer.

## 5. Conclusions

This study systematically evaluated the flame-retardant effectiveness and underlying mechanisms of highly thermally conductive copper coatings for a TC4 titanium alloy under oxygen-enriched conditions. The main conclusions are summarized as follows:

(1) The copper coating significantly enhanced the critical combustion thermodynamic thresholds of the TC4 alloy. As the coating thickness increased from 100 μm to 300 μm, the critical ignition power rose by 125–170 W compared to the substrate (235 W), the critical oxygen pressure increased by 0.21–0.51 MPa, and the ignition temperature was elevated by 119–184 K. This improvement was primarily attributed to the high thermal diffusivity of copper. The increased coating thickness reinforced heat dissipation, effectively suppressing local heat accumulation and thereby strengthening the combustion resistance.

(2) The copper coating effectively improved the combustion kinetic behavior of the material. Under identical experimental conditions, the coating extended the ignition delay time by 0.670 s and reduced the combustion propagation rate by approximately 21% (from 26.772 mm/s to 21.084 mm/s), significantly retarding the combustion process.

(3) The flame-retardant mechanism of the coating originated from a unique interfacial structure formed during combustion. Microstructural analysis revealed that the copper coating generated a TiCu_2_Al-type intermetallic compound, (Ti_0.5_Al_0.5_)Cu, which exerted an “anchoring” effect on the substrate material and reduced the Ti/O reaction efficiency, thereby achieving efficient flame retardancy.

## Figures and Tables

**Table 1:** The HVOF process parameters for the Cu coatings.

Coating	Oxygen (L/s)	Methane (L/s)	Compressed Air (L/min)	Feeding Rate (g/min)	Spraying Distance (mm)	Gun Moving Speed (mm/s)
Cu	200~310	75~120	300~400	35~45	220~280	300~400

**Table 2:** Chemical compositions of the different points from Figure 6.

Points	Element Composition (at%)				
	Ti	Al	V	Cu	O
C1	73.84	0.22	1.26	0.08	24.60
C2	30.04	23.11	8.94	33.90	0.41
C3	9.40	7.23	3.08	80.12	0.17
C4	64.48	0.73	1.18	0.10	33.51
C5	51.22	0.33	0.40	0.53	47.52
C6	16.23	7.48	67.54	8.33	0.42
C7	25.51	22.61	0.28	50.50	1.10
C8	0.98	43.95	0.53	0.50	54.04
C9	51.88	0.74	5.76	0.04	41.58
C10	38.57	2.06	3.34	0.12	55.91
C11	0.02	38.91	0.03	0.10	60.04
C12	10.54	2.48	27.23	59.43	0.32

## Data Availability

The original contributions presented in this study are included in the article. Further inquiries can be directed to the corresponding authors.
